# Ability of dairy fat in inducing metabolic syndrome in rats

**DOI:** 10.1186/s40064-016-3716-x

**Published:** 2016-11-28

**Authors:** Elham Ehrampoush, Reza Homayounfar, Sayed Hossein Davoodi, Hamid Zand, Alireza Askari, Seyed Amin Kouhpayeh

**Affiliations:** 1 Noncommunicable Diseases Research Center, Fasa University of Medical Sciences, Fasa, Iran; 2 Cancer Research Center, Shahid Beheshti University of Medical Sciences, Tehran, Iran; 3 National Nutrition and Food Technology Research Institute, Faculty of Nutrition and Food Technology, Shahid Beheshti University of Medical Sciences, Tehran, Iran

**Keywords:** Metabolic syndrome, Diet, High-calorie diet, High-fat diet, Wistar rat, HOMA-IR, Insulin

## Abstract

**Background:**

The risk of heart diseases, diabetes and stroke is increased with higher metabolic risk factors. Models of diseases resulting from high-calorie diets have a significant role in pathophysiologic analysis of metabolic syndrome in rodents; but, these diets are considerably different from each other in various studies and may not be very similar to the metabolic syndrome model in humans. This study sought to make a model close to the disease in humans. 20 five-week old male Wistar rats were randomly assigned to two groups. For one of the groups, a high-calorie diet with 416 calories per 100 g with dairy-based fat was considered and, for another group, a control diet was given for 12 weeks. Weight changes, lipid profile, glucose values, Blood pressure, insulin and HOMA indices, were measured for both groups and weight changes were compared using repeated measures and independent *t* test; also, serum results were compared using independent *t* test.

**Results:**

Values of weight, glucose, insulin, lipid profile and blood pressure, except HDL, had a tangible difference between two groups at the end of the study. HOMA-IR, HOMA-B and HOMA-S indicates a significant difference between the two groups after consumption high-energy diet.

**Conclusion:**

The present study showed ability of dairy fat in gaining weight, insulin resistance and metabolic syndrome and provided the necessity of paying serious attention to the amount of fat intake from dairy sources.

## Background

A brief look at human societies shows that most of us are overweight and it is becoming an epidemic in developed societies. Obesity can be the source of diseases like insulin resistance, abnormal levels of blood fat (high triglyceride and low high-density lipoproteins) and hypertension. The term metabolic syndrome (MS) is used to describe simultaneous occurrence of these diseases and people suffering from metabolic syndrome are at higher risk of cardiovascular diseases, type 2 diabetes, cancer and non-alcoholic fatty liver (Panchal et al. 2011; Ghaemi et al. 2013). According to the report of National Health and Nutrition Examination Survey (2003–2006), prevalence rate of metabolic syndrome in American society was still increasing by about 34% (Ford et al. 2010). It has been estimated that, in the United States, every individual with metabolic syndrome spends about 4000$ for treatment per year.

The underlying cause of metabolic syndrome is unknown although insulin resistance and visceral fat accumulation have been identified as its backgrounds (Alberti et al. 2009; Babai et al. 2016). Insulin resistance (IR) is a physiological condition in which insulin hormone finds less ability in reducing blood sugar. The subsequent increase of blood sugar can be so high that may exceed normal range of blood sugar and cause health problems. Certain cell species such as fat and muscle cells require insulin for entry of glucose inside the cell. If these cells do not respond to the insulin circulating in blood flow, blood sugar increases. Insulin resistance leaves various effects which include reduced ability of fat cells in removing blood lipids and increased hydrolysis of environmental triglycerides. This hydrolysis leads to the increased number of free fatty acids in blood flow and its subsequent health problems (Abranches et al. 2015).

Similar to many diseases, risk of metabolic syndrome is also the result of interaction between genes and the surrounding environment (Tsai and Schumann 2015). Since human genotypes have not been changed during several centuries, therefore, environmental factors should be considered the main factor of increasing suffering from metabolic syndrome in recent years (Panizzon et al. 2015). One of the major issues in this regard is energy balance which is established between energy intake through food and consumed rate of energy during physical activities. In addition to energy balance, types of food are also important. From the evolutionary perspective, it has been stated that obesity and other relevant diseases are in fact the natural result of high calorie intake (Frankenfeld et al. 2015). During evolution, since food sources were not constant and starvation periods were common, having genes which allowed for efficient storage of calorie in the form of fat was an advantage. In today’s societies, the problem is that we still have these economic genes although we are surrounded by foods rich in saturated fats, simple sugars and salt. On the other hand, there is this reality that long-term limitation of energy consumption causes increased longevity (López‐Lluch and Navas 2015).

Treatment costs of metabolic syndrome is increasing and there is no surprise that medical society is seeking animal models which simulate this disease in humans in order to test effects of certain foods on pathogenesis and/or potential human treatments on them. Owing to the axial role of diet in inducing this failure in humans, most of disease models in animals use a diet for inducing these conditions.

Animal models are systems that are made in order to create examinable conditions in other species and they can be almost divided into two categories of natural and genetic models (van der Staay 2006). Today, animals are widely used by genetic manipulation for the studies relevant to diabetes. These animals show high hyperinsulinemia that are not observed in human samples with type 2 diabetes despite its usefulness for studies. Moreover, such a genotype is not observed in humans. In contrast, the diet-induced model of diabetes caused by high-caloric diet obesity is more similar the type 2 diabetes model in humans, which is usually the result of obesity and overweight. Obese animals by diet generally have a mild increase in serum insulin level than control animals whereas their glucose level is either increased or constant (Srinivasan and Ramarao 2007; Homayounfar et al. 2015).

In this study, it was attempted to observe fat effect with diary source on capability of disease induction in addition to trying to generate the closest model of metabolic syndrome to the disease existing in the society in terms of percentage of macronutrients and energy rate.

## Methods

The protocol of the research was approved by both the Ethics Committee and the Institutional Review Board of Fasa University of Medical Sciences and was in accordance with the animal studies code (1175/1000).

20 five-week old Wistar rats were selected and randomly assigned to two groups. At the beginning of the study, weight was not significantly different in both groups (68.12 ± 4.58 vs. 72 ± 6.32, P value = 0.166). For one week, a standard diet was given to both groups in order to adapt them to the experimental conditions. At the end of the first week, one of the groups started high-calorie diet with fat from dairy sources (made by the researcher) and a control diet was given to another group. Food was presented ad libitum. The animals’ environment had temperature of 20–24 °C, 20–70% humidity and light and precise darkness cycle of 12 h. Ethical points related to animals’ maintenance and the conducted experiments in them were based on the protocol approved by animal protection organizations and university. Serum indexes were measured at the end of the study and animals’ weight was assessed in one week intervals using a scale made by Kentscientific Company with accuracy of 0.01 g.

At the end of the twelfth week, after anesthesia using ether (which has the least impact on metabolic indicators), blood was taken from the heart and animals’ serum was separated. In order to reduce chance of bias in the experiments, blood samples were coded. Lipid profile and serum insulin were measured in enzymatic manner and using kit of Kaimen Company in Elisa method, respectively. Blood pressure were determined in conscious rats from two groups by an indirect tail-cuff method (Indirect blood pressure meter BP-98A, Softron™, Tokyo, Japan).

Another applied index was HOMA indices (homeostatic model assessment) which consists of three components and shows insulin resistance (HOMA-IR), beta cell function percent (HOMA-B) and insulin sensitivity percent (HOMA-S) that calculated by HOMA-2 calculator (www.dtu.ox.ac.uk/homacalculator/).

SPSS software was used for the data analysis. In order to estimate conformity of data to normal distribution, Shapiro–Wilk test was used. Weight values were analyzed using repeated measures and independent t-test and comparing mean of serum indexes was done by independent t-test. The data were reported as mean ± standard deviation and P < 0.05 was considered significant (Tables 1, 2).

**Table 1:** Composition of high fat diet and control diet

	High fat diet			Control diet	
	Weight (%)	Energy (%)	Energy in 100 g	Energy (%)	Energy in 100 g
Carbohydrate	47	45	187	58	175
Fat	19	41	171	13	39
Protein	14.5	14	58	28	84
Total		100	416	100	302

**Table 2:** Fatty acid composition of 100 g of diet

Fatty acid	Amount (g)
C4:0	0.55
C6:0	0.41
C8:0	0.26
C10:0	0.60
C12:0	0.85
C:14:0	2.61
C:16:0	0.32
C:16:1	0.55
C:18:0	1.71
C:18:1c	3.42
C:18:1t	0.66
C:18:2c	0.17
C:18:2t	0.12
C:20:0	0.02

## Results

**Fig. 1:**
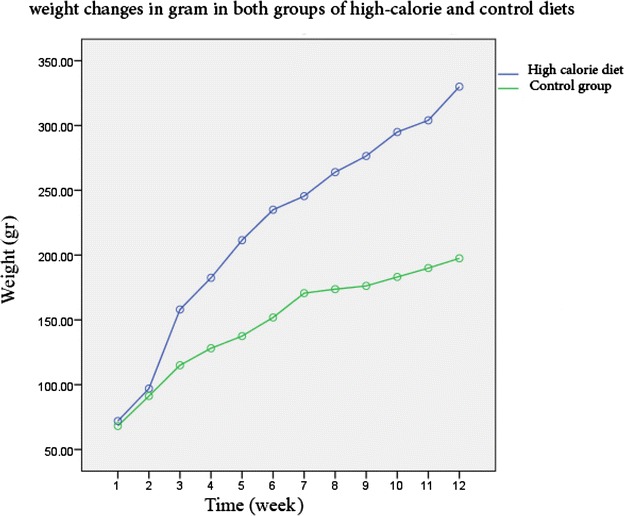
Diagram of weight changes in gram in both groups of high-calorie and control diets in 12 weeks

At the end of the twelfth week, the results were as follows. In Fig. 1, weight increase trend of both groups can be observed. The remarkable point in becoming almost flat curve of the control group since around the seventh week of starting diet which coincided with their twelfth week growth; it seems that this point was due to their maturity and stop of body growth whereas weight increase continued in the group taking the high-calorie diet.

Values of serum indexes including insulin, glucose, lipid profile and blood pressure in the group consuming high-calorie diet showed significant difference from the group consuming control diet. In fact, the difference of both groups was not significant in terms of HDL (Table 3).

**Table 3:** Serum biomarkers at the end of study

	Before	After	Differences
Insulin (ng/ml)			
High fat diet	1.97 ± 0.62	4.49 ± 1.29^b,d^	2.51 ± 1.26^b^
Control diet	1.70 ± 0.59	2.15 ± 0.46	0.44 ± 0.67
Cholesterol (mg/dl)			
High fat diet	41.74 ± 4.86	60.70 ± 6.88^b,d^	18.95 ± 7.27^b^
Control diet	40.85 ± 4.17	46.60 ± 7.82	5.75 ± 8.55
Glucose (mg/dl)			
High fat diet	145.72 ± 17.62	183.60 ± 34.90^a,c^	37.87 ± 47.54^a^
Control diet	135.27 ± 17.76	152.40 ± 15.48	17.12 ± 29.85
HDL (mg/dl)			
High fat diet	22.75 ± 7.91	34.70 ± 8.42^c^	11.94 ± 15.16
Control diet	23.27 ± 7.74	31.80 ± 10.88	8.52 ± 12.80
TG (mg/dl)			
High fat diet	53.50 ± 12.89	124.60 ± 46.43^b,d^	71.09 ± 53.84^b^
Control diet	62.43 ± 14.21	57.20 ± 24.02	−5.23 ± 28.71
Systolic BP (mmHg)			
High fat diet	128.08 ± 13.89	147.43 ± 20.84^a,c^	19.35 ± 23.89^a^
Control diet	127.21 ± 13.00	123.58 ± 16.02	−3.62 ± 23.20
Diastolic BP (mmHg)			
High fat diet	85.69 ± 8.35	94.97 ± 3.77^a,c^	9.28 ± 9.41^a^
Control diet	88.44 ± 3.98	86.86 ± 4.33	−1.57 ± 4.96
HOMA-IR			
High fat diet	0.63 ± 0.21	1.52 ± 0.24^b,d^	0.89 ± 0.21^b^
Control diet	0.65 ± 0.20	0.68 ± 0.23	0.03 ± 0.25
HOMA-B %			
High fat diet	61.27 ± 22.50	92.68 ± 18.10^a,d^	31.40 ± 29.66^b^
Control diet	60.79 ± 19.90	64.84 ± 24.73	4.04 ± 41.32
HOMA-S %			
High fat diet	67.55 ± 30.25	52.39 ± 18.97^a^	15.15 ± 34.53^b^
Control diet	72.39 ± 23.66	69.48 ± 17.00	2.90 ± 33.96

^a^Significant difference compared to control group P value <0.05

^b^Significant difference compared to control group P value <0.001

^c^Significant difference compared to baseline P value <0.05

^d^Significant difference compared to baseline P value <0.001

## Discussion

In this study, metabolic syndrome and insulin resistance were managed to be induced in normal rats (without genetic change) via dieting. As mentioned in the introduction, the term metabolic syndrome (MS) is used for describing simultaneous occurrence of conditions such as insulin resistance, abnormal level of blood fats (high triglyceride and low high-density lipoproteins) and hypertension and people suffering from metabolic syndrome are at higher risk of cardiovascular diseases, type 2 diabetes, cancer and non-alcoholic fatty liver. Considering growing trend of this disease, the importance of studying such circumstances is highlighted and, since this disease is more associated with pattern and life style of people and their tendency to use western diets, studies which can simulate such conditions and have not been made by pharmaceutical or genetic interventions will have more proximity to the society’s existing conditions; having such a model can provide the way for more studies to find cause and treatment mechanisms of these patients. Currently, genetic models of this disease are available, which have both high price and (as mentioned earlier) low generalizability to ordinary patients of the society because this disease is not normally caused by genetic defects or single nucleotide polymorphisms and is simply the result of incorrect life style, high energy consumption and inactivity. The diet which was used in this study contained 45% energy from carbohydrates, 41% from fat and 14% from protein, which was highly similar to high-energy diet used by the society. Most of commercially available diets provide 60% of fat, which is not much similar to the common diet of people due to effectiveness in obesity and inducing insulin resistance and lipid profile disorder; this issue undermines their generalizability to the population and research results. Studies have suggested that such animal models have similar characteristics to humans and they can be used for simulating human studies (Srinivasan and Ramarao 2007).

Values of diet compounds do not have a specific definition. Typically, low-fat diets contain 10% calorie from fat, high-fat diets have 30–50% energy from fat source and very high-fat diets have more than 50% energy. To induce obesity, both high-fat and very high-fat diets are used and body weight depends on the amount of fat (Ghibaudi et al. 2002).

Source of the used fat is also important. Some researchers have reported that different fats induce various phonotypes (Hariri and Thibault 2010). For instance, for rodents with equal diets in terms of fat, the ones which received fish oil gained less weight and had higher insulin sensitivity than those who had a diet rich in saturated fat (Buettner et al. 2006; Wang et al. 2002). However, all articles have not reported such results and suggest effectiveness of fat value and gender (Ikemoto et al. 1996; Pellizzon et al. 2002).

Rodents have more tendency to gain weight on high-fat or very high-fat diets. However, in glucose tolerance, insulin resistance, triglyceride levels and other factors, dependence on gender and species (Rossmeisl et al. 2003) and dietary fat sources (Buettner et al. 2006; Ikemoto et al. 1996; Wang et al. 2002) are also effective. Studies have shown that Wistar and Sprague–Dawley rats are divided into two categories of obesity resistant and getting obese with diet in terms of their response to high-fat diet. For the researchers interested in the field of obesity and type 2 diabetes, male ZDF rats are available which become obese and diabetic even by consuming low-fat diet; high-fat diet causes more serious diseases in them. Its females do not suffer from diabetes in spite of gaining weight unless they receive a diet containing 48% fat energy (Srinivasan et al. 2005). Insulin resistance induced before development of diabetes in these rats (especially, females) enable researchers to study their pre-diabetic condition (Baxter et al. 2006).

Various species of rats show different behaviors against high-fat diets (more than 60% energy from fat). Certain mice like C57BL/6 or AKR are sensitive to obesity by such diets (Rossmeisl et al. 2003); but, others such as A/J and SWR/J are resistant to obesity (Hata et al. 1996; Pereira et al. 2002). Hence, this study tried to use high-fat, not very high-fat, diets for inducing obesity and insulin resistance; additionally, generalizability to the society was attempted to be higher using this model because there are few people with energy intake of above 50%.

The first description of high-calorie diet for making obesity model dates back to 1959 (Thomas and Hartroft 1959). Its subsequent studies have demonstrated that high-fat diets cause hyperglycemia and insulin resistance (Assaad et al. 2014; Pound et al. 2014). Studies have also indicated that high consumption of saturated fat acids and cholesterol leads to increased blood cholesterol and LDL (Pound et al. 2014) and increased risk of heart diseases. Similar to humans, high-fat diet (with a large amount of saturated acids) and cholesterol (approximately about 0.2 weight percent), which is known as western diet, can cause increase in total cholesterol and LDL-C in rats.

Buettner et al. (2006) gave four types of high-fat diets with 42% energy from fat to rats which included lard, olive, coconut and fish oil in order to compare effects of various high-fat diets on metabolic processes of rats; after 12 weeks, certain indexes such as weight, insulin tolerance, lipid profile, serum glucose and insulin resistance index were measured. Their reports showed that maximum gain weight occurred in the group consuming high-fat diet with lard source, which had weight difference of about 100 g from the control group at the end of the study (504 ± 36 vs. 606 ± 54, P value <0.05). This significant difference in weight was also established for the groups consuming high-fat diet with olive oil and coconut oil sources; however, they demonstrated lower values of weight difference. In contrast, the group taking high-fat diet with fish oil source had a similar final weight to the control group. Except the group consuming high-fat with olive oil source, glucose values did not have any significant difference from the control group (5.6 ± 0.3 vs. 5.0 ± 0.3, P value <0.05). As far as insulin was concerned, there was a significant difference between the group consuming high-fat diet with lard source and the control (780 ± 230 vs. 577 ± 223, P value <0.05). Insulin resistance index in the group consuming lard fat had evident difference from the control group (27.2 ± 8 vs. 19 ± 8.5, P value <0.05).

In the study by Ghibaudi et al. (Ghibaudi et al. 2002), three diets with fat of 10, 32 and 45% energy from various sources were compared with each other. Based on the findings, high-fat diet (45% energy from fat) caused more increase of total body weight in addition to more increase in body fat percent compared with groups with medium (32%) and low (10%) fat intake (33.64% ± 1.76 vs. 28.28% ± 1.79 and 20.75% ± 2.88; P value <0.05) As the same time, it increased glucose level, cholesterol, free fatty acids, triglyceride and serum leptin compared with the mentioned groups; but, the difference of serum insulin was not significant.

In the study by Rossmeisl et al. (2003), difference of two low-fat (12% energy from fat) and high-fat (58% energy from fat) diets was investigated in two types of AKR and B6 rats. According to their reports, both groups of rats did not have any significant weight difference in both diets (low-fat and high-fat) although BS rats gained more weight increase than AKR rats. However, results were significantly different for blood glucose, insulin, triglyceride and serum free fatty acids. The study concluded that fat B6 rats had more glucose tolerance at the end of the study whereas fat AKR rats had more insulin resistance. They reported that fat tissue of B6 rats was three time higher than that of AKR rats which had GLUT4 whereas expression of this receptor in adipose tissue was similar in both rats. Thus, in addition to type of diet, it is possible to mention that class and species of animals are also determining for emergence of diseases resulting from food.

Pagliassotti et al. (2000) conducted a work in which three diets with similar calories of 68% energy from starch, 45% fat or 68% sucrose were used in four age groups of 5 (weaned), 10 (young), 18 (mature) and 58 (old) weeks old. The results showed that there was relative insulin resistance resulting from the process of aging in the comparison of groups, either at the beginning or at the end of the study; but, diet was also influential. The interesting point shown in the study was inability of diet with high sucrose in inducing insulin resistance in the weaned rats. Accordingly, the study focused on both processes of aging and diet leading to an increase in mass of body fat as a major factor for inducing insulin resistance.

To the best of the authors’ knowledge, this is the only study that has used fat from diary origin for inducing obesity and obesity related complications such as metabolic syndrome, hypertention and dyslipidemia. Although lard was not used in the present study as a fat source (which, according to most studies, is a major factor for increasing blood cholesterol and inducing insulin resistance) and animal fat with dairy source was applied, a significant increase was found in levels of glucose, insulin, cholesterol, triglyceride and also insulin resistance. In fact, no study which has used such type of fat for weight gain or insulin resistance has been found; accordingly, it can be concluded that animal fat with diary sources is capable of increasing fat and obesity as much as lard fat and can be used to substitute such fat in studies; also, such type of fat should be considered with more accuracy in dietary analyses. The importance of using fat from non-lard source in this study, is in its interoperability to Muslim cultures that religious beliefs do not permit them to use the products of pig origin. So the conventional models of diet-induced obesity, that uses Lard, could not generalize to these communities.

Numerous studies have indicated the ability of dairy fat in decreasing risk of chronic diseases, especially metabolic syndrome (Azadbakht et al. 2005; Baxter et al. 2006). CARDIA’s study showed that the diet pattern differentiated by frequent consumption of dairy products is able to reduce most risk factors of chronic diseases such as overweight (Pereira et al. 2002), hypertension (Hata et al. 1996) and hypercholesterolemia (Buonopane et al. 1992). On the other hand, there are certain studies showing that cholesterol and risk of chronic diseases may increase by consuming dairies (Lawlor et al. 2005). A point which has been neglected is fat resulting from dairy products; these studies have not paid attention to the amount of fat resulting from dairy products and have been epidemiologic studies for assessing total level of diary consumption. These positive effects might be attributed to the protein existing in dairy products or its resulting calcium. As demonstrated by the present study, if net effect of dairy fat was considered without other nutrients, ability of gain weight and insulin resistance would be evident.

Our results at first glance seem to be at odds with the results of studies that suggest a negative relationship between dairy consumption and risk of metabolic syndrome. There are even studies that have reported consuming high-fat dairy foods may decrease the risk of developing metabolic syndrome more than low-fat dairy. As an example Drehmer and colleagues (Drehmer et al. 2016) in the Brazilian population cohort (ELSA-Brazil) with a population of about 10,000 people concluded that the consumption of high-fat dairy products decreased metabolic syndrome score (−0.126 ± 0.03, P < 0.001) while low-fat dairy products showed no association. Kim and Je in a meta-analysis study (Kim and Je 2015) which included nine cohort study with a population of 35,379 people and 12 cross-sectional study with a population of 37,706 people showed that relative risk for developing the metabolic syndrome in those with the highest intake of dairy products compared to the group with the lowest intake of dairy in cohort studies was 0.85 (95% CI 0.73–0.98) and in cross-sectional studies was 0.73 (95% CI 0.63–0.86). Such results show a negative correlation between dairy consumption and the prevalence of metabolic syndrome. Chen et al. in a meta-analysis study (Chen et al. 2015) examined 15 cross-sectional study, a case–control study and seven cohort study and the results showed that higher intake of dairy products reduces the risk of metabolic syndrome by as much as 17% in cross-sectional studies and approximately 14% in cohort studies.

The point in comparing our results to these studies should be considered is in the components of the diet. Mentioned studies (Chen et al. 2015; Drehmer et al. 2016; Kim and Je 2015) indicate less chance of developing metabolic syndrome by consuming more dairy products in a normal diet while the results of our study pays to high-fat and high-energy diets impact that its energy comes from dairy fat origin. In Drehmer’s study (Drehmer et al. 2016) dietary fat intake was up 30% while the amount of fat in the diet used in our study was more than 40%. So characteristic of our study is the use of high-energy diet that origin of this added energy is from diary fat. While mentioned studies have focused more on normal consumption of dairy on a normal diet so perhaps compare our study results with them do not correct. Finally, our study was not seeking to limit the intake of dairy products in the community; rather, it was the investigative work to explore the possibility of creating a model of metabolic syndrome using a type of fat available in our society.

The relationship of fat intake from dairy with increasing insulin level, glucose and lipid profile suggests pathogenesis and risk of such types of fats. This fat which has its particular fans in the society and sometimes certain benefits are mentioned for it exposes the person to the risk of disturbing lipid balance and insulin resistance. In fact, these results can result from obesity or directly from consumption of such fats. In complimentary analysis, by adjusting for weight changes, difference of insulin values and glucose was not significant between the two groups, which indicated that effect of diet on insulin and glucose values was indirectly due to the capability of weight increase.

The HOMA values in Table 3 shows a significant insulin resistance by consuming a high calorie diet with dairy fat that emphasize on ability of dairy fat in induction of metabolic syndrome.

 In the present study, in spite of less weight increase in the group consuming high-fat diet compared to similar studies, results of glucose, insulin and lipid profile had considerable difference between the two groups and could be compared with results of other studies, which can be attributed to the nature of fat consumed in the diet (as mentioned earlier). Results of the present study can be useful for making a metabolic syndrome model for both pharmaceutical and pathological studies. We provide a disease model similar to the disease which exists in the society and indicate risk of high fat consumption with dairy sources in the society. Thus, it is necessary to recommend to people to decrease their consumption of high-fat dairies and substitute it with low-fat dairies which can nourish them with calcium, protein and nutrients existing in dairy and keep them away from facing dairy fat.

## Glossary

Metabolic Syndrome (MS): Clustering of at least three of five of the following medical conditions: abdominal (central) obesity elevated blood pressure, elevated fasting plasma glucose, high serum triglycerides, and low high-density lipoprotein (HDL) levels.
HOMA index: The homeostatic model assessment (HOMA) is a method used to quantify insulin resistance (HOMA-IR) beta-cell function (HOMA-B) and insulin sensitivity (HOMA-S).
Animal Model: Animal Model are in vivo models and are widely used to research human disease when human experimentation would be unfeasible or unethical.

## References

[CR1] Abranches MV, Oliveira FC, Conceicao LL, Peluzio MD (2015). Obesity and diabetes: the link between adipose tissue dysfunction and glucose homeostasis. Nutr Res Rev.

[CR2] Alberti K, Eckel RH, Grundy SM, Zimmet PZ, Cleeman JI, Donato KA, Fruchart J-C, James WPT, Loria CM, Smith SC (2009). Harmonizing the Metabolic Syndrome A Joint Interim Statement of the International Diabetes Federation Task Force on Epidemiology and Prevention; National Heart, Lung, and Blood Institute; American Heart Association; World Heart Federation; International Atherosclerosis Society; and International Association for the Study of Obesity. Circulation.

[CR3] Assaad H, Yao K, Tekwe CD, Feng S, Bazer FW, Zhou L, Carroll RJ, Meininger CJ, Wu G (2014). Analysis of energy expenditure in diet-induced obese rats. Frontiers Biosci (Landmark edition).

[CR4] Azadbakht L, Mirmiran P, Esmaillzadeh A, Azizi F (2005). Dairy consumption is inversely associated with the prevalence of the metabolic syndrome in Tehranian adults. Am J Clin Nutr.

[CR5] Babai MA, Arasteh P, Hadibarhaghtalab M, Naghizadeh MM, Salehi A, Askari A, Homayounfar R (2016). Defining a BMI cut-off point for the Iranian population: the Shiraz Heart Study. PLoS One.

[CR6] Baxter AJ, Coyne T, Mcclintock C (2006). Dietary patterns and metabolic syndrome-a review of epidemiologic evidence. Asia Pac J Clin Nutr.

[CR7] Buettner R, Parhofer K, Woenckhaus M, Wrede C, Kunz-Schughart L, Schölmerich J, Bollheimer L (2006). Defining high-fat-diet rat models: metabolic and molecular effects of different fat types. J Mol Endocrinol.

[CR8] Buonopane GJ, Kilara A, Smith JS, Mccarthy RD (1992). Effect of skim milk supplementation on blood cholesterol concentration, blood pressure, and triglycerides in a free-living human population. J Am Coll Nutr.

[CR9] Chen G-C, Szeto IMY, Chen L-H (2015). Dairy products consumption and metabolic syndrome in adults: systematic review and meta-analysis of observational studies. Sci Rep.

[CR10] Drehmer M, Pereira MA, Schmidt MI, Alvim S, Lotufo PA, Luft VC, Duncan BB (2016). Total and full-fat, but not low-fat, dairy product intakes are inversely associated with metabolic syndrome in adults. J Nutr.

[CR11] Ford ES, Li C, Zhao G (2010). Prevalence and correlates of metabolic syndrome based on a harmonious definition among adults in the US*. J Diabetes.

[CR12] Frankenfeld CL, Leslie TF, Makara MA (2015). Diabetes, obesity, and recommended fruit and vegetable consumption in relation to food environment sub-types: a cross-sectional analysis of Behavioral Risk Factor Surveillance System, United States Census, and food establishment data. BMC Public Health.

[CR13] Ghibaudi L, Cook J, Farley C, Heek M, Hwa JJ (2002). Fat intake affects adiposity, comorbidity factors, and energy metabolism of sprague-dawley rats. Obes Res.

[CR14] Ghaemi A, Taleban FA, Hekmatdoost A (2013). How much weight loss is effective on nonalcoholic fatty liver disease?. Hepat Mon.

[CR15] Hariri N, Thibault L (2010). High-fat diet-induced obesity in animal models. Nutr Res Rev.

[CR16] Hata Y, Yamamoto M, Ohni M, Nakajima K, Nakamura Y, Takano T (1996). A placebo-controlled study of the effect of sour milk on blood pressure in hypertensive subjects. Am J Clin Nutr.

[CR17] Homayounfar R, Jeddi-Tehrani M, Cheraghpour M, Ghorbani A, Zand H (2015). Relationship of p53 accumulation in peripheral tissues of high-fat diet-induced obese rats with decrease in metabolic and oncogenic signaling of insulin. Gen Comp Endocrinol.

[CR18] Ikemoto S, Takahashi M, Tsunoda N, Maruyama K, Itakura H, Ezaki O (1996). High-fat diet-induced hyperglycemia and obesity in mice: differential effects of dietary oils. Metabolism.

[CR19] Kim Y, Je Y (2015). Dairy consumption and risk of metabolic syndrome: a meta-analysis. Diabet Med.

[CR20] Lawlor D, Ebrahim S, Timpson N, Davey Smith G (2005). Avoiding milk is associated with a reduced risk of insulin resistance and the metabolic syndrome: findings from the British Women’s Heart and Health Study. Diabet Med.

[CR21] López-Lluch G, Navas P (2016). Calorie restriction as an intervention in ageing. J Physiol.

[CR22] Pagliassotti MJ, Gayles EC, Podolin DA, Wei Y, Morin CL (2000). Developmental stage modifies diet-induced peripheral insulin resistance in rats. Am J Physiol Regul Integr Comp Physiol.

[CR23] Panchal SK, Poudyal H, Iyer A, Nazer R, Alam A, Diwan V, Kauter K, Sernia C, Campbell F, Ward L (2011). High-carbohydrate, high-fat diet–induced metabolic syndrome and cardiovascular remodeling in rats. J Cardiovasc Pharmacol.

[CR24] Panizzon MS, Hauger RL, Sailors M, Lyons MJ, Jacobson KC, Murray Mckenzie R, Rana B, Vasilopoulos T, Vuoksimaa E, Xian H, Kremen WS, Franz CE (2015). A new look at the genetic and environmental coherence of metabolic syndrome components. Obesity.

[CR25] Pellizzon M, Buison A, Ordiz F, Ana LS, Jen KLC (2002). Effects of dietary fatty acids and exercise on body-weight regulation and metabolism in rats. Obes Res.

[CR26] Pereira MA, Jacobs DR, Van Horn L, Slattery ML, Kartashov AI, Ludwig DS (2002). Dairy consumption, obesity, and the insulin resistance syndrome in young adults: the CARDIA Study. JAMA.

[CR27] Pound LD, Kievit P, Grove KL (2014). The nonhuman primate as a model for type 2 diabetes. Curr Opin Endocrinol Diabetes Obes.

[CR28] Rossmeisl M, Rim JS, Koza RA, Kozak LP (2003). Variation in type 2 diabetes-related traits in mouse strains susceptible to diet-induced obesity. Diabetes.

[CR29] Srinivasan K, Ramarao P (2007). Animal model in type 2 diabetes research: An overview. Indian J Med Res.

[CR30] Srinivasan K, Viswanad B, Asrat L, Kaul C, Ramarao P (2005). Combination of high-fat diet-fed and low-dose streptozotocin-treated rat: a model for type 2 diabetes and pharmacological screening. Pharmacol Res.

[CR31] Thomas WA, Hartroft WS (1959). Myocardial infarction in rats fed diets containing high fat, cholesterol, thiouracil, and sodium cholate. Circulation.

[CR32] Tsai A, Schumann R (2016). Morbid obesity and perioperative complications. Curr Opin Anaesthesiol.

[CR33] Van Der Staay FJ (2006). Animal models of behavioral dysfunctions: basic concepts and classifications, and an evaluation strategy. Brain Res Rev.

[CR34] Wang H, Storlien LH, Huang X-F (2002). Effects of dietary fat types on body fatness, leptin, and ARC leptin receptor, NPY, and AgRP mRNA expression. Am J Physiol Endocrinol Metab.

[CR35] Zand H, Homayounfar R, Cheraghpour M, Jeddi-Tehrani M, Ghorbani A, Pourvali K, Soltani SR (2016). Obesity-induced p53 activation in insulin-dependent and independent tissues is inhibited by beta-adrenergic agonist in diet-induced obese rats. Life Sci.

